# Simultaneous determination of LY3214996, abemaciclib, and M2 and M20 metabolites in human plasma, cerebrospinal fluid, and brain tumor by LC-MS/MS

**DOI:** 10.1016/j.jpha.2022.05.003

**Published:** 2022-05-20

**Authors:** Tigran Margaryan, Mackenna Elliott, Nader Sanai, Artak Tovmasyan

**Affiliations:** Ivy Brain Tumor Center, Barrow Neurological Institute, Phoenix, AZ, 85013, USA

**Keywords:** LC-MS/MS, LY3214996, Abemaciclib, Brain tumor penetration, Equilibrium dialysis

## Abstract

A sensitive and rapid liquid chromatography tandem mass spectrometry (LC-MS/MS) method was established for the quantification of total and unbound concentrations of LY3214996, an extracellular signal-regulated kinase inhibitor; abemaciclib, a cyclin-dependent kinase 4/6 inhibitor; and abemaciclib active metabolites, M2 and M20, in human plasma, brain tumor, and cerebrospinal fluid samples. The method was validated over a concentration range of 0.2–500 nM within a total run time of 3.8 min using isocratic elution on a Kinetex™ F_5_ column. Detection was performed on a Sciex QTRAP 6500+ mass spectrometer employing multiple reaction monitoring mode under positive electrospray ionization. The intra- and inter-batch accuracy as well as the precision of the method for all matrices was within ±20% and ≤20% at the lower limit of quantification, and within ±15% and ≤15% for other quality control levels for all analytes. The unbound fractions of drugs and metabolites in spiked and patient samples were determined using an optimized equilibrium dialysis. The validated method was successfully applied in a phase 0/2 clinical trial to assess the central nervous system penetration of LY3214996 and abemaciclib.

## Highlights

• The LC-MS/MS method was validated to measure LY3214996, abemaciclib, M2 and M20 in human plasma, brain tumor, and CSF.
• Unbound fractions of LY3214996, abemaciclib, M2 and M20 were established in human plasma, brain, and brain tumor.
• The total and unbound levels of LY3214996, abemaciclib, M2 and M20 in glioblastoma patients have been reported.

## Graphical abstract

**Figure:**
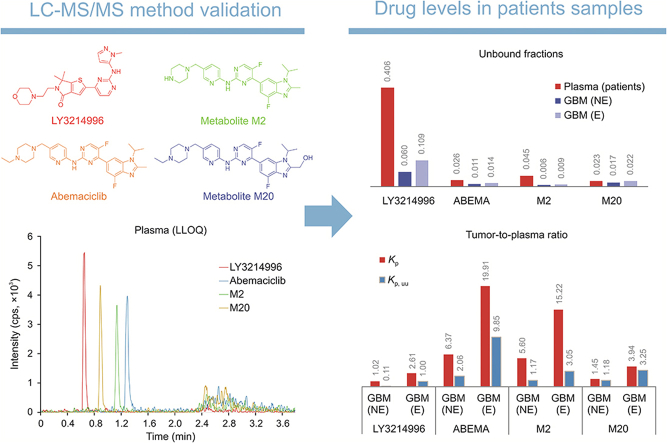

## 1 Introduction

Glioblastoma (GBM) is the most prevalent and aggressive primary malignant brain tumor in adults, with one of the worst prognoses among all cancers [1,2]. Currently available multimodal treatments, including surgery, radiotherapy and chemotherapy, deliver poor survival improvement; median overall survival is approximately 15 months after diagnosis. New innovative therapeutic strategies are continuously sought, and yet remain challenging for GBM management. This may primarily be due to tumor heterogeneity and resistance mechanisms as well as drug impermeability across the blood-brain barrier [[3], [4], [5], [6], [7], [8]].

Receptor tyrosine kinase, mainly epidermal growth factor receptor (EGFR) and platelet-derived growth factor receptor (PDGFR) signaling, and retinoblastoma (RB, 13q14)-dependent cell cycle regulation pathways are two key regulatory mechanisms controlling proliferation and cell cycle progression [9]. These two pathways converge on the cyclin-D and cyclin-dependent kinases (CDK) 4/6 regulatory complex at the G1-S phase transition checkpoint. CDK inhibitor 2A, located on 9p21 and deleted in many cancers, encodes the inhibitor of CDK4 (INK4, p16) protein, a key inhibitor of the cell cycle. Primary GBM frequently exhibits loss of the INK4A tumor suppressor gene locus along with amplifications for EGFR or PDGFR, which encode key receptor tyrosine kinases for mitogen-activated protein kinase signaling [10]. Furthermore, in GBM, the RB pathway is preferentially altered in components that lead to RB inactivation by hyperphosphorylation, thus resulting in the suppression of its ability in arresting the cell cycle. As a result, combination therapy with cell cycle and mitogenic pathway inhibitors is warranted to be evaluated. A phase 0/2 clinical trial was initiated at the Ivy Brain Tumor Center (Phoenix, AZ, USA) to investigate the potential synergism of LY3214996, an extracellular signal-regulated kinase (ERK) 1/2 inhibitor, and abemaciclib, a CDK4/6 inhibitor, in a recurrent GBM patient population (NCT04391595).

Abemaciclib, in combination with hormonal therapy, is currently approved in the US for the treatment of certain types of breast cancer [11]. It has been demonstrated that abemaciclib can cross the blood-brain barrier and inhibit tumor growth in pre-clinical orthotopic models [12]. In a recent physiologically-based pharmacokinetic modeling study, abemaciclib was suggested to be superior to other CDK4/6 inhibitors, such as ribociclib and palbociclib, for brain cancer treatment [13]. Recently, a phase II study in patients with brain metastases concluded that abemaciclib can achieve therapeutic concentrations in brain metastatic tissue, thereby warranting the exploration of novel abemaciclib-based combinations [14].

LY3214996 (temuterkib) is a newly developed potent ERK1/2 inhibitor that inhibited tumor growth in several xenograft models harboring alterations in the ERK pathway [15,16]. The agent is in at least six clinical trials that actively recruit patients with various cancers. Currently, there are no reports on the brain penetration profile of LY3214996. Our pilot pharmacokinetic studies demonstrated that the drug may cross the blood-brain barrier in mice at a pharmacologically relevant concentration.

In the present study, we aimed to develop and validate a specific, sensitive, and reliable bioanalytical method for the accurate determination of LY3214996, abemaciclib, and M2 and M20, which are equipotent abemaciclib metabolites [17], in human plasma, brain tumor, and cerebrospinal fluid (CSF). This bioanalytical method would further enable the evaluation of the total (*K*_p_) and unbound (*K*_p,uu_) brain tumor tissue-to-plasma partition coefficients for LY3214996, abemaciclib, and M2 and M20 metabolites. Herein, we report a validated liquid chromatography tandem mass spectrometry (LC-MS/MS) method for the simultaneous determination of LY3214996, abemaciclib, and M2 and M20 metabolites in the plasma, GBM tissue, and CSF of selected patients. Compared to the reported assays for abemaciclib [[18], [19], [20]], the present method demonstrated at least 10-fold better sensitivity (lower limit of quantification is 0.2 nM), a larger dynamic range (0.2–500 nM), and a minor post-injection carryover, while the sample preparation involved a simple one-step protein precipitation. To our knowledge, no validated assays have been reported for the determination of LY3214996 levels in any biological specimen. The validated method is currently being utilized in a phase 0/2 clinical trial (NCT04391595) to evaluate the total and unbound levels of LY3214996, abemaciclib, and M2 and M20 in the plasma, GBM tissue, and CSF of selected patients.

## 2 Experimental

### 2.1 Chemicals and reagents

**Fig. 1:**
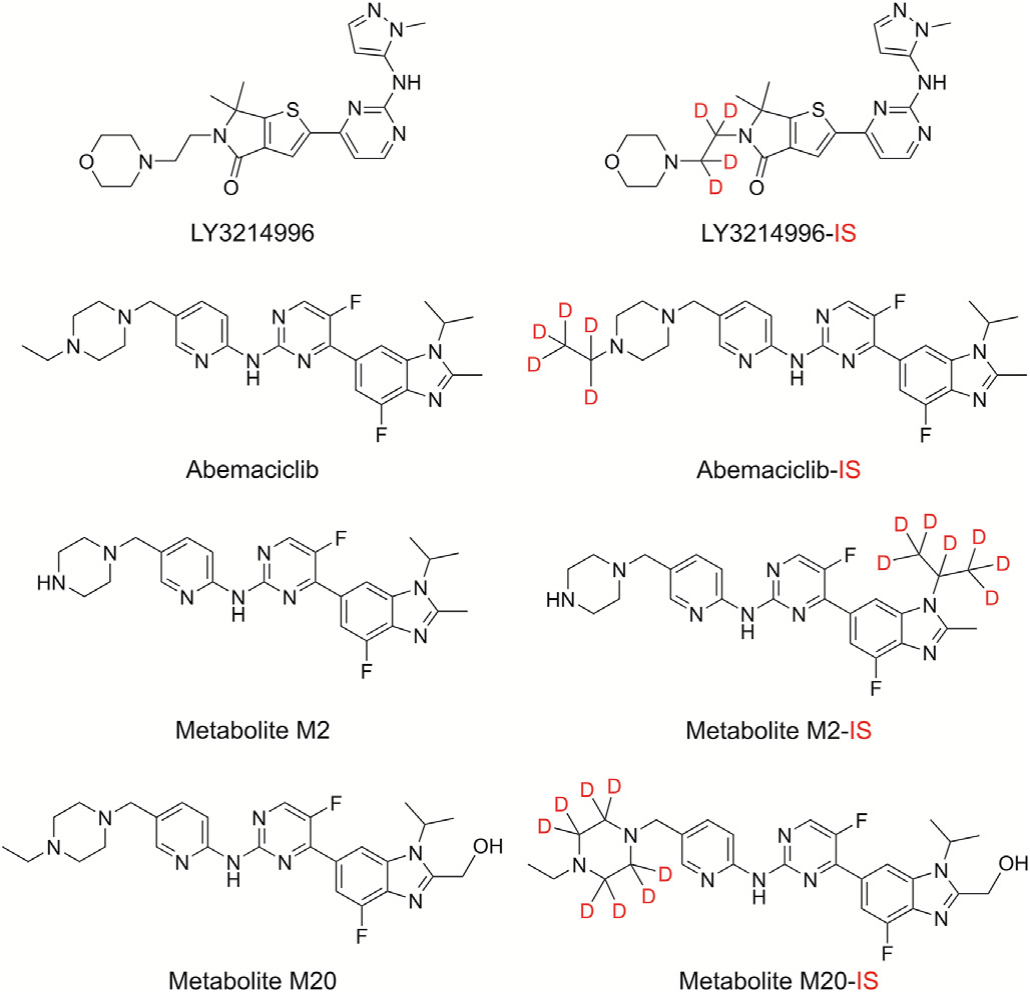
Chemical structures of LY3214996, abemaciclib, and abemaciclib metabolites M2 and M20 as well as their related stable isotope-labeled internal standard (IS).

LY3214996 (purity 98.6%), abemaciclib (purity 99.9%), M2 (mesylate salt, potency 74.9%) and M20 (mesylate salt, potency 77.5%) (Table 1 [12,21] and Fig. 1), with their stable isotope-labeled internal standards (IS) LY3214996-D_4_ (LY3214996-IS, purity 96%), abemaciclib-D_5_ (abemaciclib-IS, purity 100%), M2-D_7_ 2HCl (M2-IS, potency 86.9%), and M20-D_8_ (M20-IS, purity 90%), were provided by Eli Lilly and Company (Indianapolis, IN, USA). Phosphate-buffered saline (PBS, pH 7.4), formic acid (>98% grade), HPLC-grade ammonium formate, LC-MS-grade methanol, and acetonitrile were purchased from Fisher Scientific (Waltham, MA, USA). LC-MS-grade water was obtained from a Milli-Q IQ 7000 filter water system (Millipore Sigma, Burlington, MA, USA). The 96-well equilibrium dialyzer plates were obtained from Harvard Apparatus (Holliston, MA, USA). Human plasma (K_2_EDTA, as an anticoagulant) and CSF were purchased from Innovative Research INC (Novi, MI, USA), while the human brain was obtained from VRL Eurofins (Denver, CO, USA). The protocol for the development and validation of the LC-MS/MS method using human samples was reviewed and approved by the Institutional Biosafety Committee at St. Joseph's Hospital and Medical Center (Phoenix, AZ, USA).

**Table 1:** Physico-chemical properties of LY3214996 and abemaciclib.

Property a	LY3214996	Abemaciclib	Refs.
Molecular weight (g/mol)	453.6	506.6	
Lipophilicity (CLogP) b	2.53	4.42 (3.36) d	[12]
Distribution (LogD, pH7.4)	1.88	2.55 (2.70) d	[12]
Polar surface area (Å2)	117.0	75.0	
H-bond donors	1	1	
pKa (strongest basic) c	6.21	7.95	
Central nervous system multiparameter optimization score	4.30	3.70	[21]

a The reported properties were calculated using ACD/labs unless indicated otherwise.

b Lipophilicity, calculated as partition coefficient, CLogP, was computed using ChemDraw 18.0.

c Data were taken from investigational brochures.

d The values in parenthesis were experimentally measured.

### 2.2 Instrumentation and chromatographic conditions

**Fig. 2:**
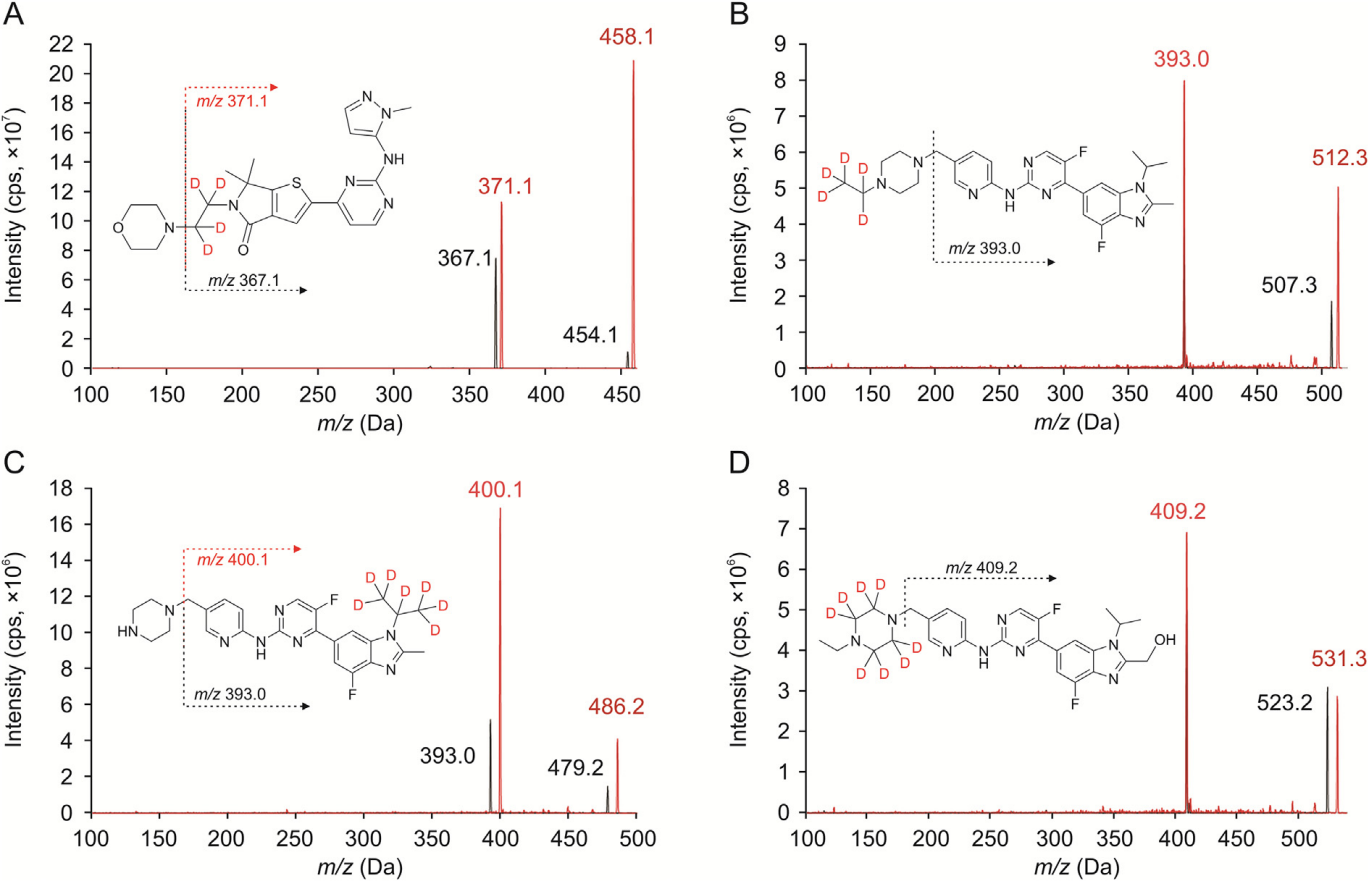
Representative product ion scan spectra of (A) LY3214996, (B) abemaciclib, (C) abemaciclib metabolite M2, and (D) abemacilclib metabolite M20 along with their related IS.

The samples were analyzed using a SCIEX ExionLC UHPLC system, coupled with Sciex QTRAP 6500+ (Foster City, CA, USA) equipped with an electrospray ionization source. Chromatographic separation was achieved at 40 °C on a Phenomenex Kinetex™ F_5_ column (100 mm × 2.1 mm, 2.6 μm; Torrance, CA, USA). Mobile phase A consisted of 5 mM ammonium formate with 0.1% formic acid (*V/V*), while mobile phase B was of acetonitrile:methanol (1:1, *V/V*). Chromatographic separation was done in isocratic mode, followed by a gradient washing step with the following sequence: 0–1.5 min 45% B (isocratic), 1.5–1.8 min 95% B (linear gradient), 1.8–2.6 min 95% (isocratic), 2.6–2.9 min 45% B (linear gradient), and 2.9–3.8 min (isocratic) at 0.5 mL/min flow rate. The total run time of the method was 3.8 min, with an injection volume of 2 μL. The autosampler temperature was 5 °C. Analyses were executed using the following parameters: ionizing voltage (5,500 V), source temperature (500 °C), curtain gas (35 psi), nebulizer gas (80 psi), and heating gas (60 psi). Multiple reaction monitoring was performed using nitrogen as the collision gas (9 psi), with a dwell time of 50 ms for each analyte and 20 ms for each IS transition. The transitions monitored for each standard and IS along with the corresponding collision energies are provided in Table 2 and Fig. 2. Data were acquired and analyzed using Analyst software version 1.7 (Foster City, CA, USA).

**Table 2:** Analyte specific MS parameters.

Parameters	LY3214996	Abemaciclib	M2	M20
MRM transition (standard, *m/z*)	454.1 → 367.1	507.3 → 393.0	479.2 → 393.0	523.3 → 409.2
MRM transition (IS, *m/z*)	458.1 → 371.1	512.3 → 393.0	486.2 → 400.1	531.3 → 409.2
Declustering potential (V)	80	80	80	80
Collision energy (V)	28	32	35	30
Collision cell exit potential (V)	20	20	20	20

MRM: multiple reaction monitoring.

### 2.3 Sample preparation

#### 2.3.1 Calibration standards and quality control (QC) samples

Stock solutions of all analytes and ISs (1 mM) were prepared in 40% acetonitrile in water; working solutions for calibration curve standards and QC samples were prepared by dilutions with 40% acetonitrile in water. The IS precipitation solution (6 nM for LY3214996-IS and M20-IS and 3 nM for abemaciclib-IS and M2-IS) was prepared by diluting the IS stock solutions with methanol. Fresh calibration standards and batch-qualifying QC samples were prepared for each batch. Bulk QC samples were prepared by spiking the working solutions with blank human plasma, CSF, or brain homogenate at a 1:19 ratio. Because of non-specific binding in CSF, blank CSF was spiked with 0.25% Tween 80 solution before adding the QC working solution at a ratio of 1:200 (*V/V*). The final concentrations of the calibration standards were 0.2, 0.5, 1, 2, 5, 20, 50, 200, and 500 nM in the plasma. Five QC levels, namely, lower limit of quantification (LLOQ QC at 0.2 nM), low (LQC at 0.6 nM), medium (MQC at 16 nM), high (HQC at 400 nM), and out-of-calibration curve (OCC QC at 2500 nM), were utilized. All stock and working solutions were stored at 4 °C, whereas QC samples were stored at −20 °C. Separate stock solutions were used to prepare the standards and QC samples.

#### 2.3.2 Plasma sample preparation

The frozen blank and patients plasma samples were thawed at room temperature. An aliquot of 70 μL of plasma was transferred into a microcentrifuge tube and 70 μL of PBS was added, followed by precipitation with 400 μL of IS-containing methanol solution. The mixture was vortexed for a few seconds and centrifuged at 12,000 r/min at 4 °C for 10 min. The supernatant was then transferred to an autosampler vial for LC-MS/MS analysis.

#### 2.3.3 Brain, brain tumor and CSF sample preparation

Blank brain and clinical brain tumor tissue homogenates were prepared at 1:9 (*m*/*V*) ratio with PBS. The samples were homogenized at a speed of 6.00 m/s for 40 s for three cycles using a Bead Ruptor Elite homogenizer (Omni International, Kennesaw, GA, USA). Plasma was used as a surrogate matrix when preparing calibration curves for brain and brain tumor homogenates and CSF as well as for the PBS fraction after sample dialysis. To minimize matrix differences, all plasma samples were spiked with PBS, while all non-plasma samples were spiked with plasma. Extraction of the analyte and IS was facilitated by protein precipitation using methanol-containing IS precipitation solution. Samples (70 μL) spiked with PBS or plasma were precipitated using 400 μL of IS precipitation solution. After centrifugation at 12,000 r/min for 10 min at 4 °C, the supernatant was transferred for LC-MS/MS analysis.

### 2.4 Equilibrium dialysis

Equilibrium dialysis was employed to measure the fraction unbound of analytes in plasma, brain, and brain tumor tissue homogenates using 96-well DispoEquilibrium Dialyzer plates with a 10 kDa cut-off regenerated cellulose membrane. Dialysis was performed using 180 μL of human plasma or tissue homogenate against an equal volume of PBS (pH 7.4) in a dual-plate rotator (Harvard Apparatus, Holliston, MA, USA). Equilibrium time was initially optimized by analyzing samples after 2, 4, 6, 8, 16, and 24 h of dialysis at concentrations of 20 and 200 nM in human plasma and brain homogenate. The dialysates were collected and processed as described in Sections 2.3.2 and 2.3.3. The recovery of analytes was calculated as the ratio of analyte concentration after dialysis (sum of concentrations found in PBS and matrix fractions) to the total concentration to ensure the reliability and reproducibility of the assay. The experiments were repeated at least three times, and each sample in triplicate and average fraction unbound values were determined.

The fraction unbound in plasma (*f*_u,__plasma_) and tissue homogenate (*f*_u,__hom_) was evaluated as the analyte concentration ratio of post-dialysis PBS (*c*_u_) to post-dialysis plasma (*c*_p_, Eq. (1)) or homogenate (*c*_hom_, Eq. (2)).

(1) $$\;f_{u,\mathrm{plasma}}\;=c_{\text{u}}\text{/}c_{\text{p}}$$

(2) $$f_{u,hom}\;=c_{\text{u}}\text{/}c_{\text{hom}}$$

The fraction unbound in the actual tissue (*f*_u, tissue_) was obtained by incorporating the unbound fraction in the tissue homogenate (*f*_u, hom_) and the dilution factor (*D*_f_) into Eq. (3).

(3) $$f_{u,tissue}\;=\;f_{u,hom}\text{/}\left[D_{\text{f}}\;-\;\left(D_{\text{f}}\;-\;1\right)f_{u,hom}\right]$$

Unbound drug levels in the actual plasma and brain tumor tissue were measured using dialysis for 16 h to reach equilibrium. The mean of fraction unbound values determined in five patient samples was calculated.

### 2.5 Bioanalytical method validation

Full bioanalytical method validation for plasma was executed to assess the sensitivity, selectivity, linearity, precision, accuracy, carryover, hemolysis effect, matrix effect, recovery, dilution integrity, and analyte and IS stability, in accordance with the guidelines provided by the U.S. Food and Drug Administration [22] and European Medicines Agency [23] for bioanalytical method validation. Partial validation of the brain homogenate and CSF was conducted to ensure the applicability of the method to these matrices.

#### 2.5.1 Sensitivity and selectivity

Six individual blank plasma lots were tested for plausible interference at the retention time of the analytes and IS. The peak areas of the analyte and IS at their related retention time in the LLOQ sample were used to calculate the interference in the blank. Any interference arising from the blank (with IS only) and double blank (without analyte and IS) must not be more than 20.0% of the peak area of the LLOQ sample at the retention time of the analyte and not more than 5.0% at the retention time of the IS.

The highest concentration of the standard (500 nM) was injected without IS in triplicate to examine possible interference at the IS retention time. An extracted solution of IS was also prepared without analytes in triplicate to monitor any interference at the related retention time. The interference from the analyte must not be more than 5.0% of the average IS peak area of the accepted calibrators and QC. The interference from IS must not be more than 20.0% of the analyte peak area of the LLOQ.

#### 2.5.2 Calibration curve, linearity, precision, and accuracy

Nine plasma samples at concentrations of 0.2, 0.5, 1, 2, 5, 20, 50, 200, and 500 nM, a blank, and a double blank were prepared to create a calibration curve. The simplest linear regression model with a 1/*x*^2^ weighting factor was utilized to define the concentration-response relationship.

At least three independent precision and accuracy tests were conducted by three chemists on different days, using two different columns for each matrix (plasma, brain homogenate, and CSF). The intra- and inter-batch accuracy (%) and coefficient of variance (CV, %) at each QC level were calculated in each batch and among batches for all analytes in plasma, brain homogenate, and CSF. Five replicates at each QC level were included in every test run. The criteria for acceptable intra- and inter-batch accuracy for both runs were set for the mean value to be within ±15.0% of the nominal value at LQC, MQC, and HQC levels, and ±20.0% at LLOQ QC. The criteria for acceptable intra- and inter-batch precision were set for the CV to be within 20.0% at LLOQ QC and within 15.0% at other QC levels.

#### 2.5.3 Carryover

The carryover effect was studied by injecting three replicates of the blank sample after the upper limit of quantification. The percentage ratio of peak areas at the retention time of each analyte in the LLOQ and blank samples was calculated; the acceptance criteria were set to not exceed 20% of LLOQ. The acceptance criteria for IS carryover were set to <5.0% of the average IS response of the accepted calibrators and QC at the IS retention time.

#### 2.5.4 Hemolysis effect

The effect of hemolysis was evaluated by utilizing an additional batch of blank matrix with 5% hemolysis. This matrix was prepared by spiking non-hemolyzed plasma with completely hemolyzed blood at 19:1 ratio. Five replicates of the LQC and HQC as well as three blank samples were analyzed.

#### 2.5.5 Matrix effect

The matrix effect was assessed using six batches of control plasma from separate donors. The matrix factor (MF) for all analytes and IS was calculated based on the ratio of the analyte peak areas in the presence (analyte spiked with post-extraction matrix) to absence of matrix (pure solution). The IS-normalized MF was determined by the ratio of the analyte MF to the IS MF. The calculation was performed in triplicate at LQC and HQC levels. The CV (%) of the IS-normalized MF determined from all matrix lots must be <15.0% to consider that no major matrix effect is present.

#### 2.5.6 Recovery

The recovery of analytes from plasma and brain homogenates was performed by assessing the peak area ratios of extracted QC (low, medium, and high) to the mean peak area ratios of post-extraction spiked samples. The latter was considered as 100% recovery; five replicates were used at each level. For analyte recovery, IS was added to the post-extraction sample to account for differences in chromatographic behavior and mass detection. The recovery of IS was estimated by assessing the ratios of IS to the analyte peak areas of five extracted samples as well as comparing to the ratios of IS to the analyte peak areas of five post-extraction spiked samples, wherein 100% recovery was accounted for. The analytes were spiked at approximately the same MQC concentration as the extracted samples. The acceptance criteria were set to ±15.0% for CV of recovery at each QC level and for IS.

#### 2.5.7 Dilution integrity

A dilution factor of 10 was evaluated for plasma and brain homogenate by diluting the OCC QC with blank plasma in five replicates. Diluted samples were prepared and analyzed along with undiluted calibration standards. In our study, 7 μL of human plasma or brain homogenate at the OCC concentration of the analytes were diluted with 63 μL of blank pooled human plasma. The precision and accuracy for the related samples were calculated; the mean value of diluted samples within ±15.0% of the nominal value and the CV within ±15.0% at each level were taken as acceptance criteria.

#### 2.5.8 Stability

The stability of the analytes was studied under various storage and processing conditions. Stability tests were performed in at least three replicates using three different tubes at two QC levels of 0.2 nM (LLOQ QC) and 400 nM (HQC) concentrations. Short- and long-term stability studies of all analytes were conducted in human blank plasma and brain homogenate under several storage conditions. Analyte stability in biological matrices was analyzed using freshly prepared calibration standards and QC samples. The percentage difference between the stability and freshly processed QC was also assessed. Re-injection reproducibility was evaluated within the sample stability period in the autosampler. Analytes were considered stable if the accuracy at each level was ±15.0% of the nominal value. Additionally, the difference from corresponding comparison QC samples must be within ±15.0%.

### 2.6 Applications

The validated bioanalytical method was employed to investigate analytes pharmacokinetics in the plasma, brain tumor, and CSF in GBM patients (ClinicalTrials.gov identifier: NCT04391595). The protocol was reviewed and approved by the Institutional Review Board of the Barrow Neurological Institute, St. Joseph's Hospital and Medical Center (Phoenix, AZ, USA). All patients enrolled in the trial provided written informed consent. Five patients were given once daily 200 mg of LY3214996 and twice daily 150 mg of abemaciclib orally for five days prior to scheduled tumor surgical removal. Blood, CSF, and GBM tissue (including gadolinium-contrast enhancing and non-enhancing regions) samples were obtained intra-operatively from each patient on the 6th day after the last dose administration on the planned operation day. Blood was centrifuged at 4 °C and 3,000 r/min for 10 min; plasma was subsequently collected. Brain tumors were resected 7–9 h after the last dose was administered. Once resected, the tissue samples were rinsed with PBS, paper dried, and freshly frozen in liquid nitrogen. The samples were then stored at −80 °C in a freezer for later analysis.

## 3 Results and discussion

### 3.1 Method development and optimization

The present bioanalytical method was developed to determine the unbound and total levels of LY3214996, abemaciclib, M2 and M20 in plasma, brain tumor, and CSF samples. A concentration range of 0.2–500 nM was chosen to detect the expected unbound and total concentrations of the analytes in all matrices. Several columns (Phenomenex Kinetex™ C_8_ (100 mm × 2.1 mm, 2.6 μm), Phenomenex Kinetex™ C_18_ (100 mm × 2.1 mm, 2.6 μm), Phenomenex Kinetex™ Biphenyl (100 mm × 2.1 mm, 2.6 μm), Phenomenex Kinetex™ F_5_ (100 mm × 2.1 mm, 2.6 μm), and Waters X-bridge Amide (100 mm × 2.1 mm, 2.6 μm)) with various mobile phase combinations were tested to achieve the desired LLOQ. The optimal chromatographic separation, along with the least carryover and the highest sensitivity for the analytes, was achieved on Phenomenex Kinetex™ F_5_ column (100 mm × 2.1 mm, 2.6 μm) using isocratic elution, with mobile phase A as 5 mM ammonium formate with 0.1% formic acid and mobile phase B as acetonitrile:methanol (1:1, *V/V*). A gradient washing step was integrated after analyte isocratic separation to prevent possible accumulation of hydrophobic compounds on the column and to minimize carryover. Precipitation with acidified methanol and acetonitrile, various washing solvents, and other washing parameters were also tested to minimize carryover. Furthermore, to maintain carryover at a minimal level with an optimal run time, a 2-μL injection volume was selected and an external needle wash was integrated before and after aspiration with methanol.

A non-specific binding effect was observed during the method validation for CSF samples. Different containers (glass, polypropylene) with various concentrations of surfactants, such as Tween 20 and Tween 80 (from 0.1% to 5% in water), were tested. The optimal condition was identified to be as 0.25% aqueous solution of Tween 80 at 1:200 spiking ratio (*V/V*) with CSF, which resulted in minimal non-specific binding. Solutions with <0.2% Tween 80 concentration resulted in lower accuracy for abemaciclib (<85%), whereas higher concentrations of Tween 80 (>0.3%) led to inaccuracy for LY3214996 (>115%). MS parameters were optimized using the designated mobile phases.

### 3.2 Method validation results

#### 3.2.1 Selectivity (blank check and interference check)

**Fig. 3:**
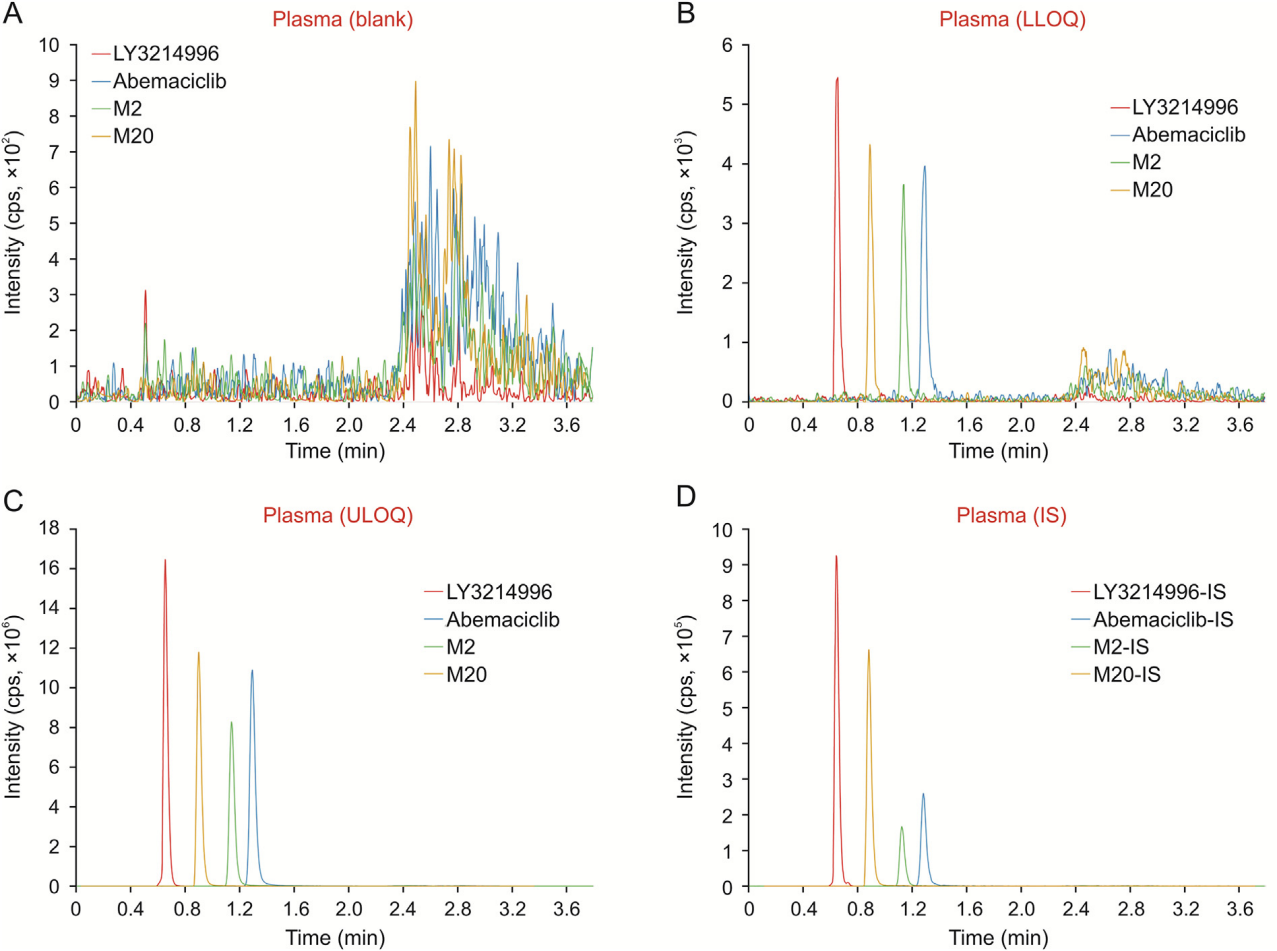
Representative ion chromatograms of (A) blank plasma samples, (B and C) plasma samples spiked with standards LY3214996 (*m/z* 454.1 → 367.1), M20 (*m/z* 523.3 → 409.2), M2 (*m/z* 479.2 → 393.0), and abemaciclib (*m/z* 507.3 → 393.0) at (B) lower limit of quantification (LLOQ), and (C) upper limit of quantification (ULOQ), and (D) plasma samples spiked with IS, i.e., LY3214996-IS (*m/z* 458.1 → 371.1), M20-IS (*m/z* 531.3 → 409.2), M2-IS (*m/z* 486.2 → 400.1) and abemaciclib-IS (*m/z* 512.3 → 393.0).

The maximum interferences at the retention time of LY3214996, abemaciclib, M2, and M20 were 2.2%, 14.4%, 14.4%, and 1.7% of LLOQ, respectively. The maximum interference at the retention time of IS was 2.0%. Typical chromatograms of the blanks, analytes, and IS are shown in Fig. 3.

#### 3.2.2 Calibration curve and sensitivity

The calibration curve was linear with a 1/*x*^2^ weighting factor over a concentration range of 0.2–500 nM. The average accuracy for calibration standards for all four analytes was between 92.8% and 104.5%, with a highest CV of 5.4%, including LLOQ. The LLOQ was established as 0.2 nM for all the four analytes (Fig. 3B). Compared to previously published assays for abemaciclib [[18], [19], [20]], the present method demonstrated at least 10-fold better sensitivity (0.2 nM versus 2 [18], 4 [20], and 200 nM [19]) and a larger linear dynamic range (2500-fold versus 100 [19,20] or 500 nM [18]). No bioanalytical method has been reported for LY3214996.

#### 3.2.3 Precision and accuracy

The results of the intra- and inter-batch accuracy and precision tests for the determination of LY3214996, abemaciclib, M2, and M20 levels in human plasma are presented in Table 3. The results for the same tests with brain homogenate and CSF are shown in Tables S1 and S2, respectively. The results met the acceptance criteria, i.e., CV were within ±20% of the LLOQ and within ±15% of other QC levels for all analytes. All QC samples met the acceptance criteria; therefore, human plasma can be utilized as a surrogate matrix for quantifying all four analytes in the brain homogenate and CSF.

**Table 3:** Precision and accuracy results in plasma.

Analyte	QC level	Nominal concentration (nM)	Intra-batch (first batch, *n* = 5)			Inter-batch			
			Mean calculated concentration (nM)	Accuracy (%)	CV (%)	*n*	Mean calculated concentration (nM)	Accuracy (%)	CV (%)
LY3214996	LLOQ QC	0.200	0.195	97.3	6.5	20	0.208	104.1	6.0
	LQC	0.600	0.568	94.7	1.6	20	0.596	99.3	6.7
	MQC	16.00	15.58	97.4	2.3	20	16.28	101.8	5.3
	HQC	400.0	398.0	99.5	1.0	20	414.8	103.7	5.6
Abemaciclib	LLOQ QC	0.200	0.214	106.8	12.0	15	0.213	106.3	0.7
	LQC	0.600	0.581	96.8	1.9	15	0.606	101.0	7.4
	MQC	16.00	16.11	100.7	1.4	15	16.76	104.8	5.6
	HQC	400.0	419.2	104.8	1.6	15	428.8	107.2	4.7
M2	LLOQ QC	0.200	0.196	97.9	4.7	20	0.217	108.4	9.7
	LQC	0.600	0.563	93.8	3.4	20	0.600	100.0	8.2
	MQC	16.00	15.80	98.7	1.7	20	16.38	102.4	5.0
	HQC	400.0	410.4	102.6	1.6	20	421.9	105.5	4.9
M20	LLOQ QC	0.200	0.203	101.7	5.0	15	0.205	102.4	5.6
	LQC	0.600	0.579	96.5	3.4	15	0.590	98.4	5.0
	MQC	16.00	15.18	94.9	1.8	15	15.97	99.8	6.0
	HQC	400.0	397.7	99.4	1.9	15	411.8	103.0	5.3

QC: quality control; LLOQ QC: lower limit of quantification QC; LQC: low QC; MQC: medium QC; HQC: high QC; CV: coefficient of variance.

#### 3.2.4 Carryover

There was no major carryover effect for LY3214996 after the 500 nM sample injection. However, we could not completely eliminate the carryover effect for abemaciclib and its metabolites. For abemaciclib and M20, carryover was insignificant after the 200 nM sample injection. Additional blank samples (at least one) is recommended to inject after the expected high concentration (>200 nM) to prevent possible repeat analysis. Otherwise, unknown samples with analyte concentrations lower than the LQC and succeeding samples with >200 nM concentration should be repeated during the clinical sample analysis. For M2, no major carryover effect was observed after the 50 nM sample injection. Based on these results, additional blank samples (at least two) should be injected after the expected high concentration (>50 nM) to prevent possible repeat analysis. Otherwise, unknown samples with analyte concentrations lower than the LQC or 2 nM and succeeding samples with >200 or >500 nM concentration, respectively, should be repeated during clinical sample analysis. Additional blank injections should be placed before predicted or known low concentrations. Carryover was 0.0% for the IS.

#### 3.2.5 Hemolysis effect

The mean accuracy results of the hemolyzed LQC and HQC samples were within 87.9%–102.3%, with the highest CV of 7.5% for all analytes. The maximum interference registered in the hemolyzed blank samples was 2.0% at the retention time of analytes and 0% at the retention time of IS. Thus, samples with <5% hemolysis can be processed for quantifying all four analytes.

#### 3.2.6 Matrix effect

The CV of the IS-normalized MF at both LQC and HQC levels was ≤6.8% for all analytes. The mean values for the IS-normalized MF ranged between 0.98–1.01, 1.02–1.06, 0.99–1.03, and 0.99–1.03 for LY3214996, abemaciclib, M2, and M20, respectively. Thus, these results suggested an insignificant matrix effect from the human plasma. The data are listed in Table S3.

#### 3.2.7 Recovery

The maximum CV of recovery samples was 8.60% for all analytes and IS in plasma and brain homogenates (Table S4). The recovery of IS and analytes at all three levels in both matrices was between 86.0% and 99.1%, except for the M2-IS. M2-IS recovery in plasma and brain homogenates was 67.1% and 55.5%, respectively. Although the difference in recovery results between M2 and M2-IS was substantial, no trend or inconsistency was recorded that could impact the accuracy of the data. Given that IS recovery was coherent and reproducible for both matrices, such a difference can be considered non-essential.

#### 3.2.8 Dilution integrity

A 10-fold dilution of human plasma and brain homogenate with human plasma was studied during validation to account for occasional high concentrations of drugs in plasma and brain tumor samples. The maximum CV value obtained for plasma was 4.3% for all four analytes, with 89.4%–98.3% accuracy range. For brain homogenates, the maximum CV value was 5.5%, with 99.2%–103.6% accuracy range for all four analytes. All the results met the acceptance criteria described in Section 2. Hence, 10-fold dilution can be applied to samples containing >500 nM for adjusting the analyte concentration within the 0.2–500 nM range without affecting the accuracy of the measured concentrations of all four analytes.

#### 3.2.9 Stability

Analyte stability in biological matrices, that is, QC samples prepared and stored for the stability period, was evaluated in comparison with freshly processed calibration standards and QC samples. The difference between stability and freshly prepared QC samples was also calculated. The stability results of the stock and working solutions for all analytes and their IS at room temperature and at 4 °C (nominal) are summarized in Table 4. All analytes remained stable in the plasma and brain homogenates after three freeze-thaw cycles. Re-injection reproducibility was also confirmed for all analytes when autosampler stability was established. The benchtop, autosampler, processed sample, and long-term stability results for all analytes are presented in Tables S5−S8 and are summarized in Table 5. The observed stability results are in agreement with previously published data on abemaciclib and its metabolites [[18], [19], [20]].

**Table 4:** Stock and working solutions stability.

Samples	Stock solution		Working solution	
	Room temperature	4 °C	Room temperature	4 °C
Analyte	17 h	120 days	15 h	120 days
Internal standard	25 h	57 days	25 h	23 days

**Table 5:** Analytes stability in human plasma and brain homogenate.

Matrix	Benchtop stability (room temperature, h)	Freeze-thaw stability	Autosampler stability (5 °C, h)	Processed sample stability (room temperature, h)	Long-term stability (−20 °C, days)
Plasma	19	3 cycles	112	23	27
Brain homogenate	6	3 cycles	96	22	36

### 3.3 Applications

The validated bioanalytical method was successfully applied to determine total drug levels in plasma, GBM tissue, and CSF, as well as to assess plasma pharmacokinetics and CNS penetration in patients enrolled in the NCT04391595 clinical trial. Representative ion chromatograms of plasma, CSF, and gadolinium non-enhancing brain tumor samples of a patient are shown in Fig. S1. The fraction unbound of all four analytes in plasma and brain tissue homogenate was measured by equilibrium dialysis within 16 h of the optimized equilibrium time (Fig. S2). Near complete (>90%) recovery for all four analytes was obtained after sample dialysis, indicating minimal non-specific binding to dialyzer plate components.

**Fig. 4:**
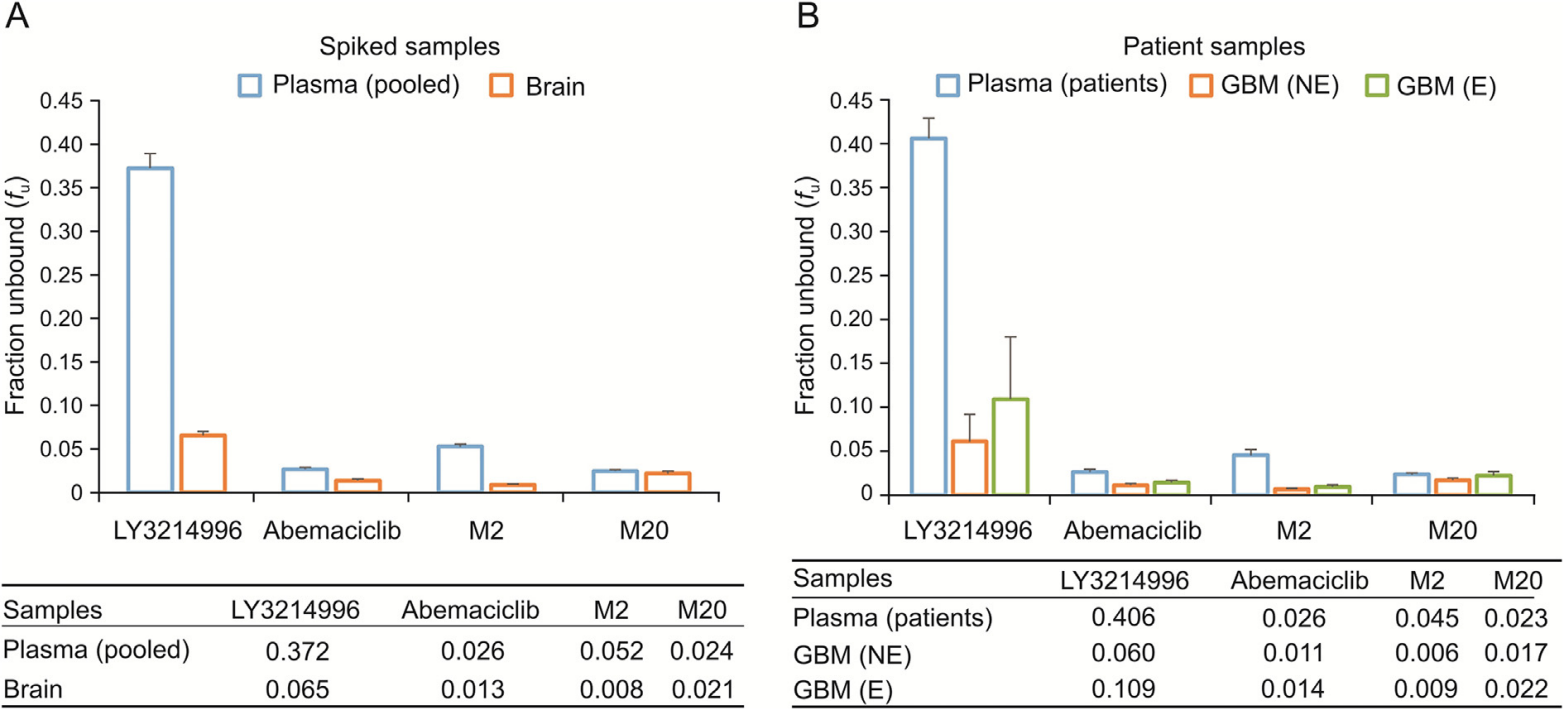
Fraction unbound of drugs and metabolites determined in (A) pooled human plasma and brain homogenate spiked with analytes and (B) plasma and glioblastoma (GBM) samples of selected patients. The 96-well microdialysis plates were used to determine the unbound concentration of analytes in the samples at an optimal 16 h equilibrium time. GBM (NE) and GBM (E) are gadolinium contrast non-enhancing and enhancing regions of GBM, accordingly, which were resected 7–9 h after the last dose administration. (A) The experiments were performed at least three times with each sample in triplicate at both 20 and 200 nM analyte concentrations. (B) The fraction unbound values were determined in five patient samples. Results are presented as mean ± SD.

Except for LY3214996, which showed modest plasma protein binding, all the other analytes showed strong binding to plasma proteins (Fig. 4A). Plasma protein binding for abemaciclib was in good agreement with a previously published *f*_u,__plasma_ value of 0.027 [12]. Binding to brain tissue components for all analytes was stronger than to plasma proteins and has not been reported previously for any of the compounds. Furthermore, LY3214996 has at least 5.5-fold higher plasma protein binding compared to brain tissue components, whereas this ratio was only 2-fold higher for abemaciclib. Interestingly, the metabolic transformation of abemaciclib appears to alter the ability of its metabolites to have non-specific interactions with brain tissue components and plasma proteins. De-ethylation of abemaciclib enhanced its binding to brain tissue components and reduced the plasma protein binding capacity of M2. As a result, M2 metabolite has at least 6.5-fold lower plasma protein binding than to brain tissue components. In contrast, hydroxylation of abemaciclib did not affect the binding of M20 metabolite to plasma proteins, and yet reduced its binding capacity to brain tissue components. In turn, M20 demonstrated a similar binding capacity to both plasma proteins and brain tissue components. Raub et al. [12] have reported the *f*_u,__brain_ values for abemaciclib as 0.0043 and 0.0079 in rat and mouse brains, which are somewhat lower than the human *f*_u,__brain_ value of 0.013.

Equilibrium dialysis for the optimum time (16 h) was employed to measure the unbound concentrations of all four analytes in the plasma and GBM samples of patients enrolled in the NCT04391595 clinical trial (Fig. 4B). In the first cohort of five patients receiving oral administration of 200 mg of LY3214996 once daily and 150 mg of abemaciclib twice daily for 5 days, the average *f*_u,__plasma_ values for both drugs and abemaciclib metabolites in plasma samples were nearly identical to the *f*_u,__plasma_ values determined with human pooled plasma samples spiked with analytes. The *f*_u,__brain_ values for LY3214996 and abemaciclib as well as the metabolites determined with the spiked human brain homogenate were between the *f*_u,__tumor_ values in gadolinium-contrast enhancing and non-enhancing brain tumor regions and were not significantly different.

The levels of drugs and metabolites were measured in intraoperatively collected CSF samples in which the analytes are considered to be primarily unbound; therefore, CSF levels are usually accounted as surrogate for unbound drug levels in brain. In five GBM patients, median levels of LY3214996, abemaciclib, M2 and M20 in CSF were 95.5 nM (range: 49.7–117.6 nM), 28.5 nM (range: 5.7–74.9 nM), 11.7 nM (range: 1.8–30.4 nM), and 12.7 nM (range: 3.1–58.1 nM), respectively.

**Fig. 5:**
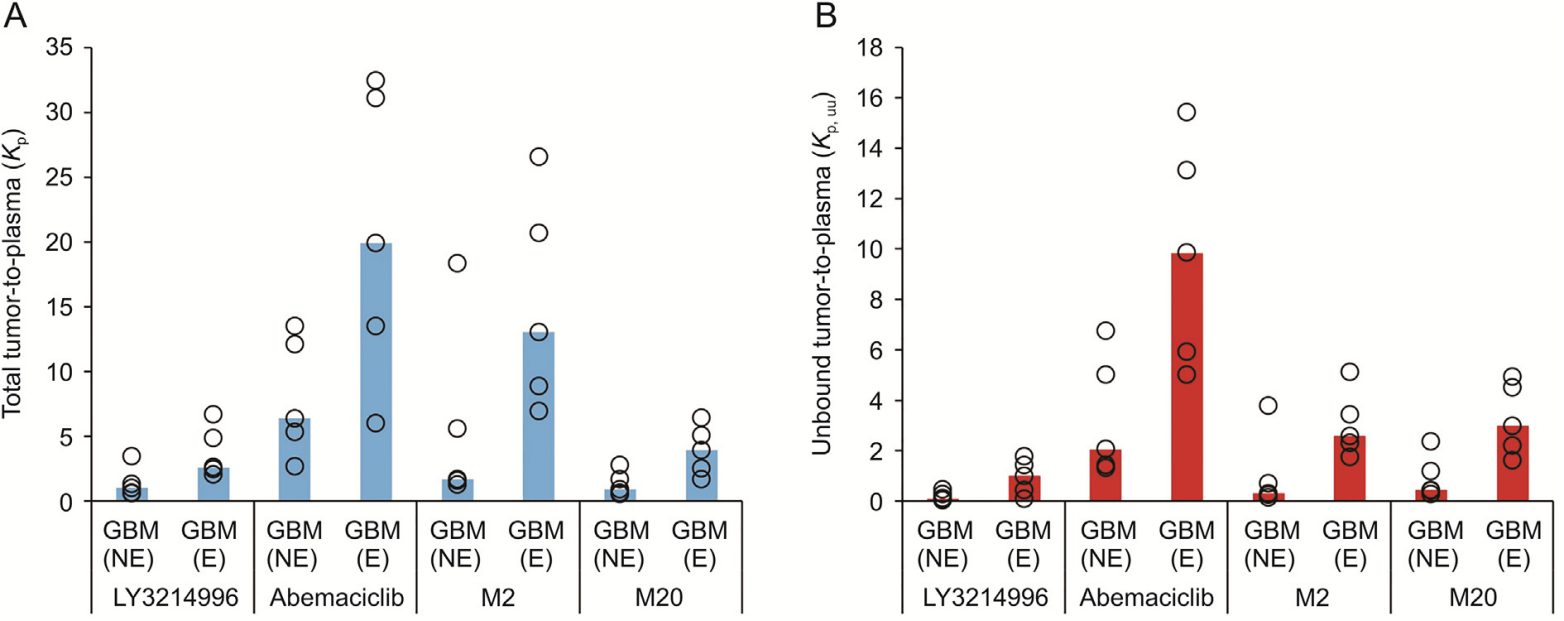
Median (A) total (*K*_p_) and (B) unbound (*K*_p,__uu_) tumor-to-plasma partition coefficients of LY3214996, abemaciclib, and abemaciclib metabolites M2 and M20 in gadolinium contrast non-enhancing (GBM (NE)) and enhancing (GBM (E)) regions of GBM, as determined in five patients. Colored bar represents the median value of tumor-to-plasma partition coefficients in five patients, while the open circle represents the individual patient result.

Median tumor-to-plasma partition coefficients for both total (*K*_p_) and unbound (*K*_p,__uu_) drug levels were determined in the gadolinium contrast non-enhancing and enhancing regions of the five GBM patients (Fig. 5). Due to stronger binding to brain tumor components than to plasma proteins, both drugs and abemaciclib metabolites possess higher *K*_p_ than *K*_p,__uu_ values. These initial clinical data show that abemaciclib had significantly higher *K*_p_ and *K*_p,__uu_ values than LY3214996 in both gadolinium contrast non-enhancing and enhancing GBM regions. This finding is contrary to what the central nervous system multiparameter optimization score had predicted based on physicochemical parameters (Table 1), that is, LY3214996 was expected to have at least as good brain penetration as that of abemaciclib. Furthermore, the obtained human *K*_p_ and *K*_p,__uu_ values for abemaciclib in the gadolinium-contrast non-enhancing GBM region, which is more representative of the normal human brain, were approximately 5 and > 12-fold higher than those reported in rodents, respectively [12]. Interestingly, both M2 and M20 metabolites had very similar unbound tumor-to-plasma partition coefficients, which were at least 3-fold lower than those of abemaciclib in both GBM regions. However, M2 had >2-fold higher total tumor-to-plasma partition coefficients in both gadolinium-contrast enhancing and non-enhancing GBM regions than M20, and better resembled the values of abemaciclib (Fig. 5). Such similarities in *K*_p,__uu_ and differences in *K*_p_ values are presumably due to altered binding properties of M2 and M20 metabolites to plasma proteins and brain tumor components.

## 4 Conclusion

The aim of this study was to establish a sensitive and rapid LC-MS/MS method for quantifying the total and unbound concentrations of LY3214996, abemaciclib, and the abemaciclib active metabolites, M2 and M20, in human plasma, brain tumor, and CSF samples. The method was fully validated with human plasma, which was also shown to function as a surrogate matrix for determining drug levels in human brain homogenate and CSF. All four analytes were detected in a single run using simple protein precipitation with methanol for a short method run time of 3.8 min. To the best of our knowledge, this is the first study to report a fully validated LC-MS/MS method for determining the total and unbound concentrations of LY3214996 in human bio-specimens. Although several methods have been previously published for abemaciclib, the present method demonstrates at least an order-of-magnitude better sensitivity, larger linear dynamic range, and minor post-injection carryover, while simple one-step protein precipitation is involved in sample processing. The validated method was successfully employed to evaluate brain tumor penetration of LY3214996 and abemaciclib in patients with GBM.

## CRediT author statement

**Tigran Margaryan**: Investigation, Methodology, Visualization, Formal analysis, Writing - Original draft preparation; **Mackenna Elliott**: Methodology, Investigation; **Nader Sanai**: Resources, Funding acquisition, Writing - Reviewing and Editing; **Artak Tovmasyan**: Conceptualization, Supervision, Methodology, Visualization, Formal analysis, Writing - Reviewing and Editing.

## Declaration of competing interest

The authors declare that there are no conflicts of interest.
