# Supplementary material for: Simultaneous determination of LY3214996, abemaciclib, and M2 and M20 metabolites in human plasma, cerebrospinal fluid, and brain tumor by LC-MS/MS
Source: J Pharm Anal. 2022 May 20;12(4):601–9. doi: 10.1016/j.jpha.2022.05.003 (PMC9463526; doi:10.1016/j.jpha.2022.05.003)
Supplement: Multimedia component 1 [file mmc1.docx]

**Supplementary Data**

**Simultaneous determination of LY3214996, abemaciclib, and M2 and M20 metabolites in human plasma, cerebrospinal fluid and brain tumor by LC-MS/MS**

Tigran Margaryan, Mackenna Elliott, Nader Sanai, Artak Tovmasyan ^*^

*Ivy Brain Tumor Center, Barrow Neurological Institute, Phoenix, AZ, 85013, USA*

^*^ Corresponding author:

*E-mail address*: [artak.tovmasyan@barrowneuro.org](mailto:artak.tovmasyan@barrowneuro.org) (A. Tovmasyan)

**Table S1.** Precision and accuracy results in brain homogenate at lower limit of quantification quality control (LLOQ QC, 0.200 nM), low quality control (LQC, 0.600 nM), medium quality control (MQC, 16.00 nM), and high quality control (HOQ, 400.0 nM) levels.

| Analyte | QC level | Intra-batch (first batch, *n*=5) | | | Inter-batch (*n*=25) | | |
| --- | --- | --- | --- | --- | --- | --- | --- |
|  |  | Mean calculated concentration (nM) | Accuracy (%) | CV (%) | Mean calculated concentration (nM) | Accuracy (%) | CV (%) |
| LY3214996 | LLOQ QC | 0.219 | 109.7 | 3.1 | 0.216 | 107.8 | 7.1 |
|  | LQC | 0.669 | 111.5 | 4.7 | 0.660 | 110.0 | 3.3 |
|  | MQC | 17.74 | 110.8 | 1.5 | 17.49 | 109.3 | 3.2 |
|  | HQC | 430.2 | 107.5 | 1.0 | 423.0 | 105.8 | 3.4 |
| Abemaciclib | LLOQ QC | 0.192 | 96.0 | 5.7 | 0.215 | 107.5 | 11.6 |
|  | LQC | 0.622 | 103.7 | 5.0 | 0.622 | 103.7 | 7.5 |
|  | MQC | 17.05 | 106.6 | 1.1 | 17.75 | 110.9 | 11.3 |
|  | HQC | 423.6 | 105.9 | 1.0 | 427.6 | 106.9 | 6.1 |
| M2 | LLOQ QC | 0.211 | 105.4 | 6.5 | 0.221 | 110.6 | 7.9 |
|  | LQC | 0.630 | 105.1 | 4.4 | 0.603 | 100.6 | 7.2 |
|  | MQC | 17.48 | 109.2 | 1.7 | 16.91 | 105.7 | 3.4 |
|  | HQC | 445.9 | 111.5 | 2.0 | 423.4 | 105.9 | 5.0 |
| M20 | LLOQ QC | 0.207 | 103.3 | 3.4 | 0.213 | 106.3 | 7.8 |
|  | LQC | 0.633 | 105.6 | 1.5 | 0.617 | 102.8 | 7.6 |
|  | MQC | 17.37 | 108.6 | 1.1 | 16.81 | 105.1 | 4.3 |
|  | HQC | 434.7 | 108.7 | 0.6 | 414.3 | 103.6 | 5.2 |

CV: coefficient of variance.

**Table S2.** Precision and accuracy results in cerebrospinal fluid at LLOQ QC (0.2 nM), LQC (0.6 nM), MQC (16 nM), and HOQ (400 nM) levels.

| Analyte | QC level | Intra-batch (first batch, *n*=5) | | | Inter-batch (*n*=20) | | |
| --- | --- | --- | --- | --- | --- | --- | --- |
|  |  | Mean calculated concentration (nM) | Accuracy (%) | CV (%) | Mean calculated concentration (nM) | Accuracy (%) | CV (%) |
| LY3214996 | LLOQ QC | 0.239 | 119.7 | 4.3 | 0.231 | 115.5 | 7.1 |
|  | LQC | 0.671 | 111.8 | 2.4 | 0.659 | 109.9 | 4.7 |
|  | MQC | 17.71 | 110.7 | 1.4 | 16.89 | 105.6 | 3.5 |
|  | HQC | 411.8 | 102.9 | 0.8 | 398.3 | 99.6 | 3.2 |
| Abemaciclib | LLOQ QC | 0.204 | 102.0 | 6.8 | 0.208 | 104.1 | 6.4 |
|  | LQC | 0.621 | 103.6 | 5.3 | 0.605 | 100.8 | 6.1 |
|  | MQC | 17.43 | 108.9 | 1.9 | 16.23 | 101.4 | 6.0 |
|  | HQC | 419.5 | 104.9 | 4.4 | 392.4 | 98.1 | 6.0 |
| M2 | LLOQ QC | 0.229 | 114.6 | 7.5 | 0.212 | 106.0 | 9.1 |
|  | LQC | 0.636 | 106.0 | 3.0 | 0.608 | 101.3 | 8.1 |
|  | MQC | 17.54 | 109.6 | 3.4 | 16.39 | 102.4 | 5.6 |
|  | HQC | 423.8 | 105.9 | 2.0 | 400.1 | 100.0 | 4.5 |
| M20 | LLOQ QC | 0.205 | 102.5 | 4.6 | 0.206 | 102.9 | 11.3 |
|  | LQC | 0.612 | 101.9 | 2.6 | 0.608 | 101.4 | 8.1 |
|  | MQC | 16.89 | 105.6 | 2.2 | 16.03 | 100.2 | 5.2 |
|  | HQC | 408.0 | 102.0 | 2.7 | 388.1 | 97.0 | 5.0 |

**Table S3.** Matrix effect test results (n=18).

| Analyte (QC level) | Parameters | MF for analyte | MF for IS | IS normalized MF |
| --- | --- | --- | --- | --- |
| LY3214996 (LQC) | Mean | 1.03462 | 1.02137 | 1.01350 |
|  | SD | 0.039 | 0.039 | 0.032 |
|  | CV (%) | 3.8 | 3.9 | 3.2 |
| LY3214996 (HQC) | Mean | 0.98597 | 1.00287 | 0.98348 |
|  | SD | 0.028 | 0.035 | 0.017 |
|  | CV (%) | 2.8 | 3.5 | 1.8 |
| Abemaciclib (LQC) | Mean | 1.22942 | 1.15465 | 1.06468 |
|  | SD | 0.081 | 0.038 | 0.059 |
|  | CV (%) | 6.6 | 3.3 | 5.6 |
| Abemaciclib (HQC) | Mean | 1.06166 | 1.04490 | 1.01640 |
|  | SD | 0.027 | 0.032 | 0.021 |
|  | CV (%) | 2.5 | 3.1 | 2.1 |
| M2 (LQC) | Mean | 1.60428 | 1.55312 | 1.03248 |
|  | SD | 0.110 | 0.079 | 0.037 |
|  | CV (%) | 6.8 | 5.1 | 3.5 |
| M2 (HQC) | Mean | 1.43642 | 1.45133 | 0.99031 |
|  | SD | 0.070 | 0.083 | 0.017 |
|  | CV (%) | 4.9 | 5.8 | 1.7 |
| M20 (LQC) | Mean | 1.15908 | 1.12929 | 1.02618 |
|  | SD | 0.065 | 0.039 | 0.041 |
|  | CV (%) | 5.6 | 3.4 | 4.0 |
| M20 (HQC) | Mean | 1.04700 | 1.05400 | 0.99361 |
|  | SD | 0.025 | 0.031 | 0.015 |
|  | CV (%) | 2.4 | 2.9 | 1.5 |

MF: matrix factor; IS: internal standard.

**Table S4.** Recovery test results.

| Analyte | Plasma recovery (%) | | | | Brain recovery (%) | | | |
| --- | --- | --- | --- | --- | --- | --- | --- | --- |
|  | LQC | MQC | HQC | IS | LQC | MQC | HQC | IS |
| LY3214996 | 87.5 | 91.2 | 92.8 | 99.1 | 92.0 | 96.1 | 92.1 | 94.8 |
| Abemaciclib | 86.7 | 92.1 | 91.7 | 87.3 | 88.0 | 95.3 | 94.0 | 86.0 |
| M2 | 87.6 | 89.5 | 89.7 | 67.1 | 91.4 | 93.8 | 90.9 | 55.5 |
| M20 | 87.0 | 90.7 | 92.6 | 86.5 | 93.0 | 94.2 | 95.0 | 86.0 |

**Table S5.** LY3214996 stability results at LOQ (0.6 nM) and HOQ (400.0 nM) levels under different conditions.

| Matrix | Parameter | Benchtop stability | | Freeze-thaw stability | | Autosampler stability (5 °C) | | Processed sample stability | | Long-term stability (−20 °C) | |
| --- | --- | --- | --- | --- | --- | --- | --- | --- | --- | --- | --- |
|  |  | LQC | HQC | LQC | HQC | LQC | HQC | LQC | HQC | LQC | HQC |
| Plasma | Period | 19 h | | 3 cycles | | 112 h | | 23 h | | 27 days | |
|  | Mean concentration (nM) | 0.586 | 409.3 | 0.606 | 402.5 | 0.555 | 381.8 | 0.547 | 390.7 | 0.609 | 418.1 |
|  | Accuracy (%) | 97.7 | 102.3 | 101.0 | 100.6 | 92.5 | 95.4 | 91.1 | 97.7 | 101.5 | 104.5 |
|  | CV (%) | 1.2 | 1.1 | 3.1 | 1.9 | 1.9 | 1.1 | 1.7 | 0.9 | 1.1 | 0.6 |
|  | Difference (%) | 3.2 | 2.8 | 5.3 | 3.2 | 3.1 | 1.5 | −3.4 | 2.2 | −2.2 | 3.4 |
| Brain homogenate | Period | 6 h | | 3 cycles | | 96 h | | 22 h | | 39 days | |
|  | Mean concentration (nM) | 0.661 | 406.7 | 0.608 | 412.9 | 0.667 | 420.9 | 0.674 | 409.3 | 0.622 | 422.7 |
|  | Accuracy (%) | 110.2 | 101.7 | 101.4 | 103.2 | 111.2 | 105.2 | 112.3 | 102.3 | 103.6 | 1.0 |
|  | CV (%) | 4.4 | 2.4 | 4.5 | 0.9 | 0.9 | 0.3 | 1.7 | 1.9 | 1.3 | 105.7 |
|  | Difference (%) | 0.2 | −1.5 | −7.9 | 1.1 | 4.4 | 3.3 | 2.1 | −0.9 | −5.9 | 3.5 |

**Table S6.** Abemaciclib stability results at LOQ (0.6 nM) and HOQ (400.0 nM) levels under different conditions.

| Matrix | Parameter | Benchtop stability | | Freeze-thaw stability | | Autosampler stability (5 °C) | | Processed sample stability | | Long-term stability (−20 °C) | |
| --- | --- | --- | --- | --- | --- | --- | --- | --- | --- | --- | --- |
|  |  | LQC | HQC | LQC | HQC | LQC | HQC | LQC | HQC | LQC | HQC |
| Plasma | Period | 19 h | | 3 cycles | | 112 h | | 23 h | | 27 days | |
|  | Mean concentration (nM) | 0.613 | 424.7 | 0.578 | 422.6 | 0.547 | 418.6 | 0.558 | 432.2 | 0.568 | 444.3 |
|  | Accuracy (%) | 102.2 | 106.2 | 96.3 | 105.7 | 91.2 | 104.7 | 93.1 | 108 | 94.6 | 111.1 |
|  | CV (%) | 2.5 | 2.5 | 5.2 | 3.3 | 2.9 | 1.3 | 1.5 | 1.2 | 6.5 | 1.2 |
|  | Difference (%) | 5.6 | 1.3 | −1.3 | 2.4 | 2.4 | −1.3 | −2.8 | 3.3 | −4.8 | 4.9 |
| Brain homogenate | Period | 6 h | | 3 cycles | | 96 h | | 22 h | | 39 days | |
|  | Mean concentration (nM) | 0.65 | 389.0 | 0.626 | 427.3 | 0.65 | 411.2 | 0.627 | 398.4 | 0.619 | 434.7 |
|  | Accuracy (%) | 108.3 | 97.3 | 104.4 | 106.8 | 108.3 | 102.8 | 104.5 | 99.6 | 103.1 | 108.7 |
|  | CV (%) | 2.8 | 3.6 | 4.3 | 2.4 | 1.7 | 1.3 | 2.5 | 1.8 | 7.4 | 2.6 |
|  | Difference (%) | 6.7 | −3.7 | −5.7 | −3.2 | 13.8 | 3.3 | 3.0 | −1.4 | −6.9 | −1.5 |

**Table S7.** M2 stability results at LOQ (0.6 nM) and HOQ (400.0 nM) levels under different conditions.

| Matrix | Parameter | Benchtop stability | | Freeze-thaw stability | | Autosampler stability (5 °C) | | Processed sample stability | | Long-term stability (−20 °C) | |
| --- | --- | --- | --- | --- | --- | --- | --- | --- | --- | --- | --- |
|  |  | LQC | HQC | LQC | HQC | LQC | HQC | LQC | HQC | LQC | HQC |
| Plasma | Period | 19 h | | 3 cycles | | 112 h | | 23 h | | 27 days | |
|  | Mean concentration (nM) | 0.553 | 415.0 | 0.632 | 424.2 | 0.581 | 390.9 | 0.573 | 402.2 | 0.6 | 437.0 |
|  | Accuracy (%) | 92.2 | 103.8 | 105.3 | 106 | 96.8 | 97.7 | 95.5 | 100.6 | 99.9 | 109.2 |
|  | CV (%) | 0.4 | 2 | 2.3 | 2.9 | 2.8 | 3.1 | 5.6 | 0.2 | 7.4 | 0.6 |
|  | Difference (%) | −1.7 | 1.1 | 5.8 | 5.8 | 4.8 | −0.2 | 3.6 | 0.7 | −3.1 | 5.6 |
| Brain homogenate | Period | 6 h | | 3 cycles | | 96 h | | 22 h | | 39 days | |
|  | Mean concentration (nM) | 0.614 | 388.2 | 0.590 | 405.1 | 0.615 | 421.6 | 0.632 | 400.9 | 0.590 | 413.6 |
|  | Accuracy (%) | 102.4 | 97.0 | 98.3 | 101.3 | 102.4 | 105.4 | 105.4 | 100.2 | 98.3 | 103.4 |
|  | CV (%) | 4.3 | 5.1 | 5.4 | 3.0 | 4.135 | 1.3 | 0.3 | 1.8 | 1.4 | 1.8 |
|  | Difference (%) | 6.9 | −5.9 | −9.4 | −6.8 | 13.5 | 6.2 | 10.0 | −2.8 | −9.5 | −4.8 |

**Table S8.** M20 stability results at LOQ (0.6 nM) and HOQ (400.0 nM) levels under different conditions.

| Matrix | Parameter | Benchtop stability | | Freeze-thaw stability | | Autosampler stability (5 °C) | | Processed sample stability | | Long-term stability (−20 °C) | |
| --- | --- | --- | --- | --- | --- | --- | --- | --- | --- | --- | --- |
|  |  | LQC | HQC | LQC | HQC | LQC | HQC | LQC | HQC | LQC | HQC |
| Plasma | Period | 19 h | | 3 cycles | | 112 h | | 23 h | | 27 days | |
|  | Mean concentration (nM) | 0.599 | 408.9 | 0.583 | 398.6 | 0.57 | 382.5 | 0.534 | 405.7 | 0.61 | 418.8 |
|  | Accuracy (%) | 99.9 | 102.2 | 97.2 | 99.6 | 95.1 | 95.6 | 88.9 | 101.4 | 101.7 | 104.7 |
|  | CV (%) | 2.2 | 0.5 | 3.6 | 2.6 | 2.7 | 0.5 | 3.6 | 0.6 | 4.2 | 1.2 |
|  | Difference (%) | 3.5 | 2.8 | 2.9 | 0.3 | 1.3 | −2 | −2 | 2.7 | −4 | 2.7 |
| Brain homogenate | Period | 6 h | | 3 cycles | | 96 h | | 22 h | | 39 days | |
|  | Mean concentration (nM) | 0.6 | 375.0 | 0.651 | 434.9 | 0.612 | 401.3 | 0.603 | 391.1 | 0.635 | 448.1 |
|  | Accuracy (%) | 100.0 | 93.7 | 108.5 | 108.7 | 101.9 | 100.3 | 100.5 | 97.8 | 105.9 | 112.0 |
|  | CV (%) | 5.9 | 5.6 | 5.2 | 2 | 2.7 | 2.9 | 3.0 | 2.2 | 6.4 | 3.6 |
|  | Difference (%) | 2.3 | −4.4 | −1.5 | 1.4 | 10.3 | 2.7 | 2.7 | −0.2 | −3.9 | 4.5 |


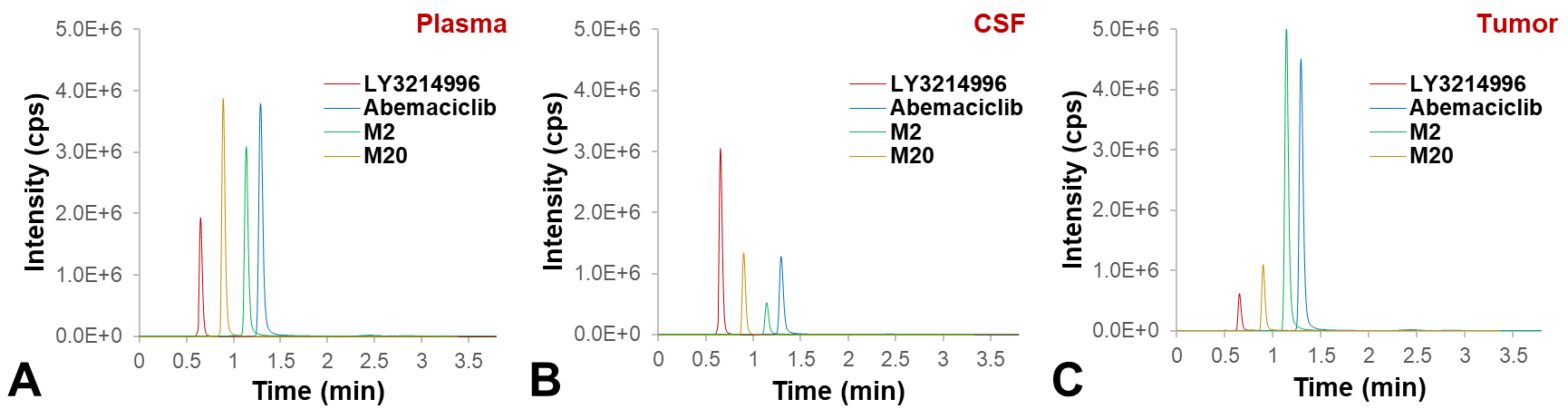


**Fig. S1.** Representative ion chromatograms of a patient (A) plasma, (B) cerebrospinal fluid, and (C) gadolinium non-enhanced brain tumor samples collected intra-operatively at 8 h after the last dose of oral administration of LY3214996 (200 mg, once daily) and abemaciclib (150 mg, twice daily) for 5 days. Appropriate mass transitions of LY3214996, M20, M2, and abemaciclib are given in the Fig. 3.


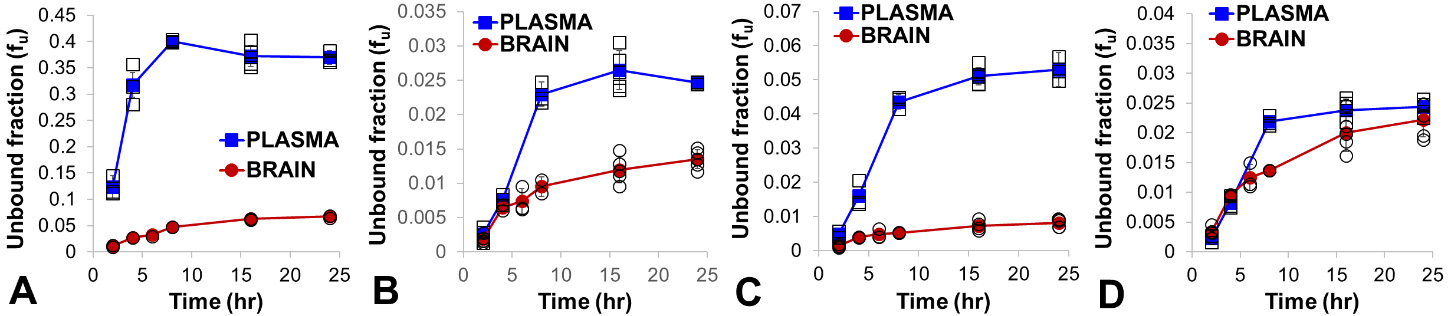


**Fig. S2.** Fraction unbound of (A) LY3214996, (B) abemaciclib, and abemaciclib metabolites (C) M2 and (D) M20 in pooled human plasma and brain determined at the equilibrium time of 2, 4, 6, 8, 16, and 24 h. Filled markers, which are connected with solid lines, represent the mean ± SD of triplicate measurements in at least 3 separate experiments. The analyte concentrations used for the equilibrium dialyses were 20 and 200 nM in pooled human plasma and brain homogenate. There was no significant difference between the concentrations at various time points tested for both human plasma and brain homogenate.
